# ICD-11 and mental disorders: important changes, controversies, and future directions

**DOI:** 10.1186/s12888-023-05186-w

**Published:** 2023-09-25

**Authors:** Florian Seemüller

**Affiliations:** 1 Department for Psychiatry, Psychosomatics and Psychotherapy, kbo-Lech-Mangfall-Klinik Garmisch-Partenkirchen, Auenstrasse 6, Garmisch-Partenkirchen, 82467 Germany; 2 Department for Psychiatry and Psychotherapy, Ludwig-Maximilians-University Munich, Nussbaumstrasse 7, Munich, 80336 Germany

## Abstract

About a year and a half after publishing ICD-11, we aim to gather initial feedback, comments, opinions, and even recent study results from experts in the relevant fields through this collection. We hope to facilitate a preliminary summary of whether the new classification truly represents progress, and how it has changed treatment, and research of mental illnesses.

## Main text

The first version of the international Classification of Diseases (ICD) has been introduced 130 years ago. The ICD-1 focused primarily on mortality statistics, serving to standardize the causes of death reported by different countries. This new classification was developed in 1893 and allowed for more accurate tracking and comparison of disease burdens across nations and continents, contributing to the early understanding of public health patterns and trends [1].

A significant change during the last transition from ICD-9 to ICD-10 was the progression from “melancholia” and “reactional depression” in ICD-9 to ‘major Depression’ in ICD-10. This step was based on research that emerged in the 1980s, showing that depressive episodes exist on a continuum between mild and severe or acute and chronic. Moreover, the evidence suggested a multifactorial origin including various factors such as genetics, hormone levels or comorbid medical conditions. In addition to that there was no difference in response to a pharmacological antidepressant treatment [2]. More recent research suggests that there are only few distinguishing features and a large overlap between depression subtypes [3].

After almost 30 years and more than a decade development WHO published a prefinal version of the ICD-11 in June 2018 and the final version officially on 11 February 2022. Its latest development included the participation of a global clinical practice network including nearly 15,000 clinicians from 155 countries. The major goals of the revision of Chap. 6: “Mental, behavioural or neurodevelopmental disorders” include scientific validity, globally applicability, the inclusion of the cultural context of each disorder, clinical usefulness and the further harmonization of DSM-5 and ICd-11 [4]. Currently, 35 countries are using ICD-11, translations into German, French, Arabic and Chinese are already available, but many countries like Germany still rely on ICD-10 and are preparing the introduction.

One major change is the avoidance of arbitrary cut-offs for diagnostic symptoms but description of essential features that occur nearly in all cases to closely resemble clinical practice. Another important new feature is standardized information for each chapter defining boarders to normal behavior and to differential diagnoses [5, 6]. Whereas the old chapter structure of ICD-10 relied on Kraepelin’s original textbook, the structure of ICD-11 follows a developmental perspective starting with neurodevelopmental disorders and ending with dementia. Some of the important changes include new disorders. For example, the sexual health chapter now corresponds to the idea that gender variant behavior and preferences are not sufficient for making a diagnosis. Other new disorders include catatonia, bipolar disorder type II, body dysmorphic disorder, olfactory reference disorder, hoarding disorder, excoriation disorder as well as the already in ICD-10 existing hypochondriasis (health anxiety disorders), which now is placed under the new lone standing chapter obsessive-compulsive and related disorders (see Table 1).

**Table 1:** New disorders introduced in Chap. 6 of ICD-11

New Disorder	ICD-11 Chapter
Catatonia	Catatonia
Bipolar disorder type II	Mood disorders/Bipolar or related disorders
Body dysmorphic disorder	Obsessive or related disorders
Olfactory reference disorder	
Hoarding disorder	
Excoriation disorder	
Hypochondriasis	
Complex posttraumatic stress disorder	Disorders t associated with stress
Prolonged grief disorder	
Binge eating disorder	Feeding or eating disorders
Avoidant/restrictive food intake disorder	
body integrity dysphoria	Disorders of bodily distress or bodily experience
gaming disorder	Disorders due to addictive behaviors
compulsive sexual behavior	Impulse control disorders
intermittent explosive disorder	
premenstrual dysphoric disorder	Depressive disorders

Regarding existing diagnoses, the criteria for a **major depressive** episode have undergone minimal changes. They now consist of three symptom categories: affective symptoms, neurovegetative symptoms, and cognitive symptoms. Among these, five symptoms must be present for most of the day, nearly every day, for a period of two weeks. Here, a cut off has been maintained. Moreover, a dimensional approach to categorize symptom severity and the use of specifiers to indicate clinical characteristics such as psychotic features or melancholic symptoms has been introduced [6]. Fortunately, the ICD-11 retained the so-called “bereavement exclusion” from the ICD-10, albeit in a somewhat mitigated form. Therefore, a diagnosis of a depressive episode in connection with a grief reaction can only be made if it lasts longer (at least 4 weeks) and if it includes certain typical depressive symptoms such as suicidal ideation, feelings of guilt, low self-esteem, psychotic symptoms, and psychomotor retardation. This is where the distinction from normal mental experience becomes particularly interesting. Already at the introduction of DSM-5, I expressed in a critical comment that the elimination of the boundary between a depressive episode and a “healthy” grief reaction is likely to lead to pathologizing of normal mental experiences and thereby further degrade the specificity of a major depression diagnosis [7]. Current research findings have now confirmed that a wrong path was taken in DSM-5. In long-term studies the risk of developing a second depressive episode was significantly lower in people with a grief associated episode as compared to patients with a major depressive episode. Moreover, this risk was just as low as in people who never experienced a depressive episode before [8–10].

### Bipolar disorders

remain a chapter within mood disorders in the ICD-11, as opposed to their classification in the DSM-5. The major changes largely involve harmonization between the DSM-5 and ICD-11. A manic episode can now only be diagnosed when increased activity is present and a minimum duration of seven days has been established. In addition, bipolar II disorder has been included. Unlike the DSM-5, which uses a specifier for mixed symptoms, the ICD-11 now allows diagnosing mixed episodes.

### Anxiety disorders

now include “separation anxiety disorder” and “selective mutism” underlining its life span approach. There is no distinction between “panic disorder” and other “anxiety disorders” anymore. Within the chapter “disorders specifically associated with stress”, an “acute stress reaction” now is understood as normal reaction to an extreme stressor and is therefore otherwise classified within the chapter “factors influencing health status or contact with health services”. Within the chapter of “dissociative disorders” the term conversion has been eliminated and “dissociative neurological symptom disorder” is now a single disorder with twelve different subtypes.

In **anorexia nervosa** the threshold for low body weight has been increased form 17,5 kg/m2 to 18,0 kg/m2 and the requirement of a widespread endocrine disorder has been eliminated.

A major shift has also been undertaken in the diagnosis of **personality disorders**. While a borderline pattern qualifier remains, all other personality disorders have been replaced by specifications on one or more of the five trait domains: negative affectivity, detachment, dissociality, disinhibition and anakastia.

Within disorders due to **substance use or addictive behaviors** the ICD-11 includes the new category of ‘alcohol harmful use,‘ which captures cases where alcohol consumption leads to physical or mental harm but may not meet the criteria for alcohol dependence.

### Dementia

underwent a major shift, while the manifestation of different dementias like Alzheimers and Lewy-body dementia still can be encoded under Chap. 6, the diagnose moved from the mental disorders chapter in ICD-10 to the new chapter on ‘Neurological Disorders’ in ICD-11. This relocation recognizes dementia as primarily a neurological condition, rather than a mental disorder.

Though the original Kraepelinian categorization of the chapters was abandoned in the ICD-11, one of his central insights remains extremely relevant to the diagnosis of psychiatric disorders today: the greatest precision and certainty in diagnosis is particularly evident when observed over the long-term course of the psychiatric illness. This is why it is not only important that health systems enable continuity of care in the psychiatric field, but also that every clinician is aware of the relative nature of psychiatric diagnoses. This requires an open approach to diagnostic uncertainties and good communication with the patient about potential differential diagnoses, pointing out the long-term course of the disease. An overly hasty and incorrect diagnosis carries significant risks such as unnecessary self-stigmatization and chronicity due to a delay in the correct diagnosis and the accompanying treatment.

Now, about a year and a half after publishing ICD-11, we aim to gather initial feedback, comments, opinions, and even recent study results from experts in the relevant fields through this collection. With this, and in view of the recently published text revision of DSM-5, we hope to facilitate a preliminary summary of whether the new classification truly represents progress, and how it has changed the diagnosis, treatment, and research of mental illnesses for better or worse.

## Data Availability

Not applicable.

## List of abbreviations

ICD: International Classification of Diseases
DSM: Diagnostic and Statistical Manual of Mental Disorders
WHO: World Health Organization
